# A SNP based linkage map of the turkey genome reveals multiple intrachromosomal rearrangements between the Turkey and Chicken genomes

**DOI:** 10.1186/1471-2164-11-647

**Published:** 2010-11-20

**Authors:** Muhammad L Aslam, John WM Bastiaansen, Richard PMA Crooijmans, Addie Vereijken, Hendrik-Jan Megens, Martien AM Groenen

**Affiliations:** 1 Animal Breeding and Genomics Centre, Wageningen University,6709PG, Wageningen, The Netherlands; 2 Hendrix Genetics, Research & Technology Centre, 5830 AC, Boxmeer, The Netherlands

## Abstract

**Background:**

The turkey (*Meleagris gallopavo*) is an important agricultural species that is the second largest contributor to the world's poultry meat production. The genomic resources of turkey provide turkey breeders with tools needed for the genetic improvement of commercial breeds of turkey for economically important traits. A linkage map of turkey is essential not only for the mapping of quantitative trait loci, but also as a framework to enable the assignment of sequence contigs to specific chromosomes. Comparative genomics with chicken provides insight into mechanisms of genome evolution and helps in identifying rare genomic events such as genomic rearrangements and duplications/deletions.

**Results:**

Eighteen full sib families, comprising 1008 (35 F1 and 973 F2) birds, were genotyped for 775 single nucleotide polymorphisms (SNPs). Of the 775 SNPs, 570 were informative and used to construct a linkage map in turkey. The final map contains 531 markers in 28 linkage groups. The total genetic distance covered by these linkage groups is 2,324 centimorgans (cM) with the largest linkage group (81 loci) measuring 326 cM. Average marker interval for all markers across the 28 linkage groups is 4.6 cM. Comparative mapping of turkey and chicken revealed two inter-, and 57 intrachromosomal rearrangements between these two species.

**Conclusion:**

Our turkey genetic map of 531 markers reveals a genome length of 2,324 cM. Our linkage map provides an improvement of previously published maps because of the more even distribution of the markers and because the map is completely based on SNP markers enabling easier and faster genotyping assays than the microsatellitemarkers used in previous linkage maps. Turkey and chicken are shown to have a highly conserved genomic structure with a relatively low number of inter-, and intrachromosomal rearrangements.

## Background

The turkey (*Meleagris gallopavo*, MGA) is an important agricultural species that is largely used as a meat type bird. In 2008, turkey represented 6.65% of the world poultry meat production [1]. The world-wide turkey population has rapidly grown due to increased commercial farming. Global turkey stocks nearly tripled from 178 million in 1970 to over 482 million in 2008. Over the same time period, the production volume increased more than fivefold from 1.2 to 6.1 million tons [1].

The turkey genome consists of 39 pairs of autosomes and 1 pair of sex chromosomes [2]. The predicted size of the turkey genome is 1.1 billion bases on the turkey genome build UMD 2.01 which is based on sequences from a combination of two next generation sequencing platforms, Roche 454 and Illumina GAII with 5× and 25× coverage respectively. Limited information is available on the turkey linkage and physical map although a small number of low resolution linkage maps using microsatellite markers [2,3] have been published. Linkage maps of chicken on the other hand are more abundant and have generally used larger numbers of markers [4-10]. Comparative cytogenetic and linkage maps between turkey and chicken showed conserved synteny and close ancestral relation among these species [2,3] and support the hypothetical ancestral Galliform karyotype [11]. Chromosome banding and zoo-FISH with chromosome paints for the turkey and chicken chromosomes have suggested that chicken and turkey karyotypes are distinguished by at least two interchromosomal rearrangements [2,12,13]. Chicken chromosome 2 and 4 are represented by turkey chromosomes 3 and 6 and by turkey chromosomes 4 and 9 respectively [2,3,13].

Chromosome studies have revealed that the karyotype is more conserved among avian lineages than it is among other groups, such as mammals, with most avian species showing a diploid chromosome number between 76 and 80 http://www.genomesize.com. This suggests that chromosomal evolution or large-scale rearrangements affecting chromosome number occur at a low rate in birds and as a result many chromosomes have remained more or less intact during avian evolution [14]. Chicken chromosome specific probes have been used for in situ hybridization onto metaphase spreads of other birds and revealed an overall picture of a high degree of chromosomal homology between chicken and representatives from many avian orders [15]. Hybridization results also indicated that interchromosomal rearrangements have been rare during avian evolution [16,17].

A linkage map is essential for the mapping of quantitative trait loci (QTL) and very useful for the assembly of genome sequence and subsequently mapping of genes along the chromosomes. A high-resolution linkage map facilitates fine mapping of quantitative trait loci (QTLs) and can be produced because of the abundance of SNPs within the genome [18]. SNP based genotyping is preferred because it is highly accurate, quick and automated, using limited human intervention. Increasing the marker density of the linkage map further enables the analyses of genomic sequences associated with high recombination rates [9].

The present study was designed to develop a SNP based linkage map in turkey and to detect genomic rearrangements between turkey and chicken.

## Methods

### Experimental population

Parents were randomly selected from two different lines to produce F1 offspring. Ten parent males were randomly selected from a line that was selected for high growth and ten parent females were randomly selected from a line that was selected for high reproduction. Average body weight of males in the high growth line from which ten parent males were randomly selected was 20.6 Kg at 20 weeks of age and the average egg production of females in high reproduction line from which ten parent females were randomly selected was 115.5 hatching eggs/24 weeks. An F2 generation of 18 full sib families was produced by crossing 17 randomly selected F1 males and 18 randomly selected F1 females. One male was mated with two females, other F1 parents were mated only once. In total, 973 F2 offspring were produced with an average full sib family size of 54.1 with a range from 31-90 individuals. All families were used for the SNPs genotyping to construct linkage maps of different chromosomes.

### DNA isolation

Genomic DNA was isolated from blood samples collected in 10% EDTA using either the automated nucleic acid extraction CAS-1820 X-tractor Gene (Corbett Life Science), or the manual nucleic acid extraction using Gentra Puregene Blood Kit (Qiagen) following manufacturer's protocol with minor modifications.

DNA concentrations were measured using ND-1000 Spectrophotometer (NanoDrop) and diluted to the required concentration of 50 ηg μL^-1^.

### SNP selection

Previously, we identified 11,287 SNPs in turkey by sequencing reduced representation libraries on an Illumina GA sequencer [19]. To achieve an even spacing of SNPs across the 40 turkey chromosomes while a turkey genome sequence was not available, SNPs in turkey were selected based on their orthologous position on the chicken genome sequence (WASHUC2 build, May 2006). Currently the chicken genome [20] covers 30 of the 39 chromosomes in chicken which comprises approximately 95% of chicken genome. By this approach, we did not select SNPs in parts of the turkey genome that are syntenic to genomic regions in chicken that are currently not represented in the chicken genome assembly. Assembled turkey short read contigs from Kerstens et al. [19] that contained SNPs were mapped on the chicken genome. Short read contigs in the size range of 50-100 bp were mapped using Megablast [21] and short read contigs of 100 bp and longer were mapped using BlastZ [22] with contig alignment criteria of at least 80% alignment and at least 60% sequence identity. In total 6,537 SNPs could be assigned a syntenic location on the chicken genome. In addition to chicken genome location, the final selection criteria for SNPs also included the Illumina design score and the estimated minor allele frequency based on the Illumina sequences from Kerstens et al [19]. The distance (in bps) between the selected SNPs was varied based on the size of the chromosome, because of the higher recombination frequency on the microchromosomes of birds. Chicken chromosomes were divided into three groups; 1-10 + Z, 11-19 and 20-28 + LGE22 and the average SNP spacing chosen for these three groups was 1.4-1.9 SNP per Mb, 0.7-1.0 SNP per Mb and 0.4-0.6 SNP per Mb respectively.

In addition, seven SNPs derived from 5 different turkey genes i.e. *Pit1*, *AFABP*, *PRKAG3*, *IGF2 *and *GDF8 *were also used.

### Genotyping

Two 384-plex GoldenGate oligo pool assay (OPA) sets were designed for genotyping using VeraCode technology on an Illumina BeadXpress Reader. The GoldenGate assay was performed according to manufacturer's protocol and as described in Fan et al. [23] and Hyten et al. [24]. Automated genotype clustering and calling was performed with GenomeStudio™data analysis software (Illumina). All genotype calling results were manually checked and any obvious errors in calling the homozygous or heterozygous clusters were corrected.

SNPs selected from the 5 turkey genes (*Pit1*, *AFABP*, *PRKAG3*, *IGF2 *and *GDF8*) were genotyped with an ABI SNaPshot assay and analyzed on an ABI 3730 DNA Analyzer (Additional file 1).

### Genetic Linkage analysis

Genotyping data was filtered by removing uninformative markers, markers giving Mendelian errors in more than one families and markers with low call rate as described by Groenen et al. [9]. The modified CRI-MAP software version 2.4 [25] by Xuelu Liu (Monsanto), which can handle much larger numbers of markers segregating in complex pedigrees was utilized for the linkage analysis.

Map building was performed step by step using AUTOGROUP, BUILD, CHROMPIC, FLIPSN, and FIXED options of CRI-MAP according to the procedures used by Stapley et al. [26] and Elferink et al. [10]. Using AUTOGROUP, parameter layers utilized for getting linkage groups were as follows: layer 1 (20, 0, 2, 0.3); layer 2 (20, 0, 20, 0.3); layer 3 (10, 0, 20, 0.3) and layer 4 (5, 0, 20, 0.3). Layer 4 had minimum stringency with likelihood ratio (LOD score) >5, 0 times the average number of meiosis, shared linkages with not more than 20 groups and with 0.3 of minimum linkage ratio [25]. Linkage groups were assigned to specific turkey chromosomes using the already known physical positions of turkey SNPs in the chicken genome and comparative information from the cytogenetic study of Griffin et al. [2] on turkey and chicken. Turkey chromosome names were assigned using the nomenclature used by Griffin et al. [2].

Maps are reported as sex averaged maps unless otherwise indicated and map figures were drawn with the MapChart software version 2.2 [27].

### Comparative genetic analysis

The order of SNPs on our linkage map was compared to the expected order based on the turkey and chicken genome assemblies UMD 2.01 and WASHUC2, respectively. Positions on the chicken genome were obtained earlier in the SNP selection step. Positions on the turkey genome were obtained by aligning SNP flanking sequences (< 1.0 × E^-4 ^) using BLAST with megablast option [28] against the turkey reference genome sequence (UMD 2.01).

The turkey physical map order of SNPs was used to validate the linkage map order with CRI-MAP using the BUILD option. The order of SNPs in linkage maps was modified if the physical map order had a higher likelihood and total chromosome map length was smaller than the linkage map order. The genetic distance between the terminal markers of every chromosome from the turkey linkage map was compared to the genetic distance between the corresponding positions of the chicken genome. First, the sequence positions (bp) of these terminal turkey markers were found on the chicken physical map. Second, chicken markers were taken from the study of Elferink et al. [10] at the closest position (bp) to these sequence positions (bp). Finally the genetic distance between these chicken markers was calculated and compared to the turkey map length.

### Analysis of recombination rate and sequence motif densities

The physical distance (Mb) on turkey chromosomes was calculated between the first and the last SNP of the linkage map using the blastall option in BLAST [28]. Number of Mb covered by the linkage map (cM) was used to calculate recombination rate (cM/Mb) for every turkey chromosome which was compared to the physical size (Mb) of the chromosomes [9,26]. The recombination rates (cM/Mb) were also compared to those for the chicken chromosomes described by Elferink et al. [10].

Densities of sequence motifs/elements CCCCCCC, CCTCCCT, CTCTCCC, CpG and CTCF consensus sequence CCNCCNGGNGG were found to vary with chromosome in chicken [9], therefore we also calculated these densities for each turkey chromosome from the turkey genome sequence (UMD 2.01). Only the part of the chromosome sequence covered by the linkage map was used to calculate these densities. Number of elements per Mb was calculated and compared against chromosome length (cM) except for CpG that was compared against cM/Mb [9].

### Ethical approval for the use of animals in this study

Although animals were used in this experimental work, no direct experiments were performed on them. Blood sample collection was carried out by licensed and authorized personnel under approval of Hendrix Genetics. No approval from the ethics committee was necessary.

## Results

### Genotyping results

Genotyping call rates with an average of 0.80 were obtained. In total, 775 SNPs (2× 384-plex GoldenGate + 7 additional SNPs) were selected for genotyping and out of these, 98 SNP assays failed (missing genotypes in the whole population), 80 SNPs appeared to be monomorphic (AA, or BB genotype) or positive for parologous sequences (all genotypes AB), 13 SNPs showed non-Mendelian inheritance in more than one family and 14 SNPs had zero informative meiosis. In total 205 SNPs were removed from the dataset.

### Linkage maps

After filtering of genotyping data, 570 SNP markers were left for the linkage analysis. Of the total 570 markers that met all quality criteria, 531 markers were found significantly linked which were subsequently inserted at their most likely position (BUILD option, LOD > 3) on one of 28 linkage groups that subsequently were assigned to 27 autosomes and the Z chromosome (Table 1). The number of informative meiosis for a marker varied from 7 to 666 with an average of 255. The largest chromosome, MGA1, had a map with 81 SNPs and a map size of 325.8 cM, followed by MGA2 with 55 SNPs and a map size of 229 cM. The chromosomes MGA25 and MGA30 had the lowest number of SNPs (4 each) as well as the smallest map sizes with map lengths of 23.5 and 6.3 cM respectively (Table 1). The total length of the sex average map (excluding the Z chromosome) was 2,165 cM and the average marker spacing was 4.4 cM. Sex specific analysis showed a difference in the male and the female maps. For 70% of chromosomes, male maps were longer than female maps, except for chromosomes MGA10, 11, 15, 16, 23, 25, 26 and MGA28 where the female maps were longer (Table 1). In general, a difference in length of 9% was observed between sex specific maps.

**Table 1:** Comparison of maps of turkey and chicken chromosomes based on genetic and physical sizes.

	**Turkey**					**Chicken**		
	
**Chromosome**	**Number of SNPs**	**Female (cM)**	**Male (cM)**	**Average (cM)**	**Length (Mb)**	**Chromosome**	**Genetic length (cM)**	**Syntenic region (Mb)**
	
MGA1	81	318.9	344.7	325.8	200.7	GGA1	353.4	193.3
MGA2	55	216.4	249.9	229	115.1	GGA3	233.5	111.7
MGA3	40	140.6	149.5	140.9	89.7	GGA2q	149.5	134.7
MGA4	27	94	146.7	120.9	67.4	GGA4q	132.1	70.0
MGA5	33	108.1	131.3	118.3	59.8	GGA5	116.2	61.2
MGA6	23	98.9	111.9	104.4	48.2	GGA2p	111.9	131.7
MGA7	22	65.3	77.3	71.4	29.6	GGA7	102.5	37.5
MGA8	19	64.1	67.4	67.3	32.2	GGA6	82.5	33.0
MGA9	10	52.9	59.7	55.2	16.8	GGA4p	60.5	91.3
MGA10	21	82.1	65.7	76.7	30.0	GGA8	56.0	29.7
MGA11	19	64.1	54.6	59.8	22.8	GGA9	78.0	22.3
MGA12	14	56.6	63.1	58.4	14.1	GGA10	45.6	18.8
MGA13	17	51.2	59.8	54.4	18.0	GGA11	62.9	20.9
MGA14	13	50.7	59.9	55.1	14.0	GGA12	44.3	13.7
MGA15	21	59	56.9	59	16.1	GGA13	56.2	17.4
MGA16	13	46.7	41.1	40.7	12.0	GGA14	47.5	12.1
MGA17	13	57.5	59.5	57.5	12.5	GGA15	52.7	12.3
MGA19	10	49.9	57.6	51.2	8.9	GGA17	48.7	10.0
MGA20	12	54.6	67.3	60.6	9.3	GGA18	48.7	9.3
MGA21	12	54.5	76	60.8	8.9	GGA19	41.9	8.3
MGA22	10	53.8	60.3	56.2	11.3	GGA20	42.0	10.6
MGA23	9	53.2	50.6	61.4	4.5	GGA21	41.1	4.9
MGA24	4	26	38.6	33.1	1.9	GGA22	21.6	1.8
MGA25	4	25.2	22.2	23.5	4.3	GGA23	30.8	4.8
MGA26	8	71.3	50.7	57.3	6.0	GGA24	51.8	5.7
MGA28	8	60.2	45	52.6	4.2	GGA26	45.5	4.3
MGA30	4	1.4	4.9	6.3	1.1	GGA28	17.1	0.9
Total autosomal	522	2077.2	2272.2	2164.8	859.4	Total	2174.5	1072.2
MGAZ	9	---	159.1	159.1	80.02	GGAZ	221.9	74.3
	
Total	531	2077.2	2431.3	2323.9	939.4	Total	2396.4	1146.5

### Comparative genetic results

#### Marker order

For all except three of the turkey chromosomes the comparison of the linkage and the physical maps did not reveal any differences. For the three chromosomes, MGA2, 11 and 17, the marker order from the physical maps, showed a higher likelihood and a smaller map distance than the marker order obtained from our linkage analyses. Log likelihood values for MGA2, 11 and 17 were increased by 20.6, 98.6 and 0.7 and map distance reduced by 4.0, 17.0 and 1.3 cM respectively. For these three chromosomes the marker order based on the physical map was used in further analyses.

Marker orders were found to be highly conserved between the turkey linkage and the chicken physical maps although 57 rearrangements were still detected between these species. The order of the SNP markers on chromosomes MGA14, 21, 25, 26 and MGAZ even showed 100% accordance with the order in the syntenic chicken chromosomes (Additional file 2).

The linkage maps for the turkey and the chicken chromosomes generally showed small differences in their lengths. Three exceptions are turkey chromosomes MGA1, MGA7 and MGAZ that showed a difference of more than 25 cM with their syntenic chicken chromosomes GGA1 GGA7 and GGAZ. Whole genome genetic map size of chicken was 72.5 cM larger than the whole genome genetic map size of turkey. In the comparisons of genetic lengths of turkey and chicken chromosomes, the difference in the reference genome positions (bp) of turkey SNPs genotyped in the present study and the genome positions (bp) of chicken SNPs used in the study by Elferink et al. [10] were small. On average the distance between the reference positions was 58,614 bp which will have caused an average difference of 0.28 cM/chromosome based on the average figure of 4.8 cM per Mb in Turkey. The total physical map size of turkey covered by markers genotyped in this study was 939.4 Mb. This is smaller than the region of the chicken physical map covered by the turkey genetic map which is 1146.5 Mb (Table 1).

#### Rearrangements

**Figure 1:**
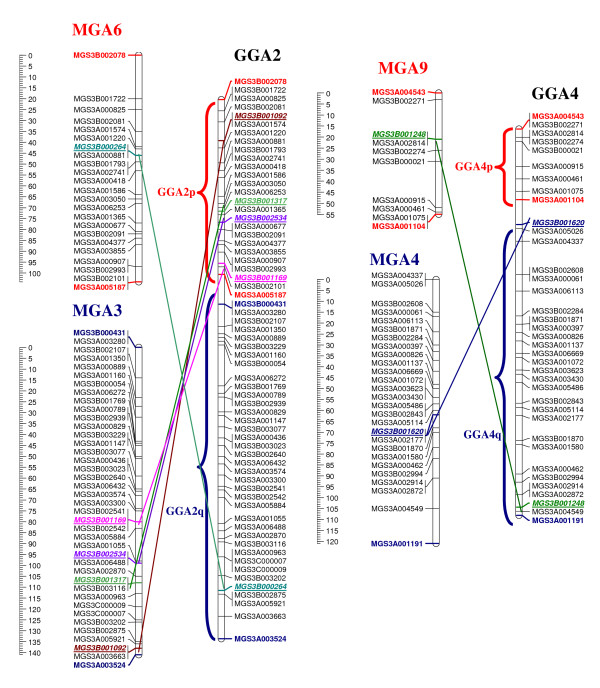
**Inter and intra chromosomal rearrangements between turkey and chicken**. Linkage maps of turkey chromosomes MGA3, MGA6 and MGA4, MGA9 showing inter and intra chromosomal rearrangements (fission, fusion and inversions,) with their syntenic chicken chromosomes GGA2 and GGA4 (maps based on physical position of SNPs in chicken genome).

Two interchromosomal and 57 intrachromosomal rearrangements were observed between turkey and chicken (Figure 1). Two linkage groups, MGA3 and MGA6 were obtained from the SNPs selected with syntenic positions on chicken chromosome 2 and similarly two linkage groups, MGA4 and 9 were obtained from the SNPs selected from chicken chromosome 4 (Figure 1). These chromosomes (MGA3, 6 and MGA4, 9 Vs GGA2 and GGA3 respectively) did not only show interchromosomal rearrangements, but also showed multiple intrachromosomal rearrangements between turkey and chicken (Figure 1).

**Figure 2:**
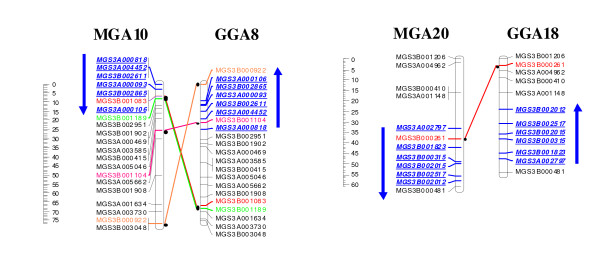
**Intrachromosomal rearrangements between turkey and chicken involving nearly a complete chromosome arm**. Turkey chromosomes MGA10 and MGA20 (genetic linkage maps) showing intrachromosomal rearrangements (Inversions) compared to the syntenic chicken chromosomes GGA8 and GGA18 (maps based on physical position of SNPs in chicken genome).

Regions with inverted marker order were observed on turkey chromosomes 10 and 20 when compared to their syntenic chicken chromosomes GGA8 and GGA18 (Figure 2). Other complex intrachromosomal rearrangements were also observed on turkey chromosome 1, 2, 5, 8, 11, 12, 13, 15, 16, 17, 19, 22, and 28 when compared to their syntenic chicken chromosomes (Additional Files 2 &3).

The number of rearrangements per Mb varied considerably for different chromosomes. The average number of rearrangements per Mb for larger chromosomes (MGA1-MGA10) was 0.06, ranging from 0.01-0.13 with highest rate of rearrangements of 0.13 per Mb on MGA10. The average number of rearrangements per Mb for the smaller chromosomes (MGA11-MGA30) was 0.11, ranging from 0.08 - 0.42 with highest rate of rearrangements of 0.42 per Mb on MGA12.

Comparative analysis of the turkey linkage, the turkey physical and the chicken physical maps showed discordance in the chromosomal allocation of 6 SNPs to these maps (Table 2). The turkey linkage and the chicken physical maps agreed with each other in the chromosomal allocation of these 6 SNPs while the turkey physical map disagreed. For example, according to the turkey linkage and chicken physical maps the SNP MGS3A000968 was assigned to MGA1 and GGA1 while this SNP was positioned on MGA8 in the turkey physical map (Table 2). Fourteen SNPs could not be assigned to any position on the turkey physical map while the allocation of these 14 SNPs to the turkey linkage map and the chicken physical map also agreed with each other (Table 2).

**Table 2:** SNPs with discordance in allocation on turkey genome with turkey genetic and chicken physical map.

SNP_ID	Turkey Linkage map (MGA)	Turkey physical map (MGA)	Chicken physical map (GGA)
MGS3A000968	1	8	1
MGS3A003050	6	13	2
MGS3A000053	8	19	6
MGS3A004543	9	10	4
MGS3A000578	15	14	13
MGS3B002546	19	8	17
MGS3A005799	1	NA	1
MGS3B003240	2	NA	3
MGS3A007335	2	NA	3
MGS3A005026	4	NA	4
MGS3B000939	5	NA	5
MGS3A007520	5	NA	5
MGS3A006539	7	NA	7
MGS3A007601	15	NA	13
MGS3A007553	16	NA	14
MGS3A002797	20	NA	18
MGS3C000006	1	NA	1
MGS3C000009	3	NA	2
MGS3B001450	Z	NA	Z
MGS3B002754	Z	NA	Z

SNP chromosomal assignment by turkey linkage, turkey sequence and chicken sequence maps NA = Not Aligned

### Recombination rate and sequence elements

**Figure 3:**
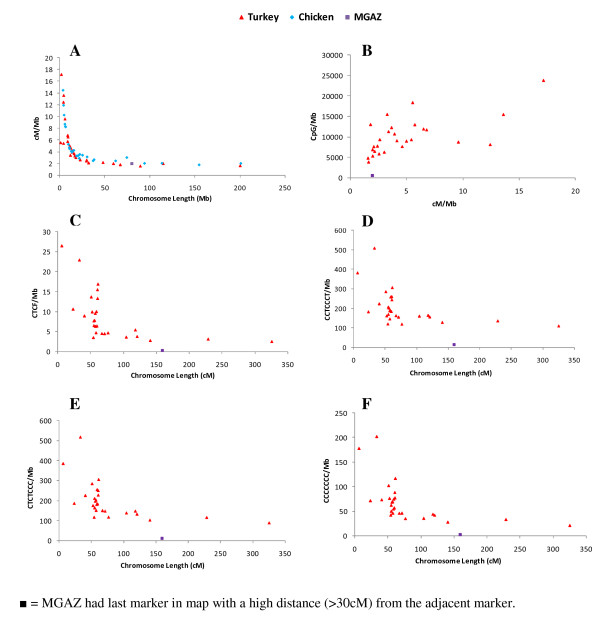
**Distribution of recombination rates and sequence motif densities across the turkey chromosomes**. Recombination rate cM/Mb was correlated with chromosome length (Mb) and CpG/Mb was correlated with recombination rate (cM/Mb). All remaining sequence motifs (CCCCCCC, CCTCCCT, CTCF and CTCTCCC) were correlated with chromosome length (cM).

Recombination rate of turkey chromosomes varied from 1.6 to 17.2 cM/Mb. The physical length of chromosomes showed an inverse relation with recombination rate while CpG/Mb density across the chromosome showed a direct relation. Turkey and chicken chromosomes of smaller sizes showed higher recombination rates than chromosomes with larger sizes (Figure 3A). CpG content showed increasing values with increasing recombination rate, i.e. higher CpG content in smaller chromosomes (Figure 3B). The frequency of sequence elements (CTCF, CCTCCCT, CTCTCCC and CCCCCCC) per Mb was found to be negatively correlated with the genetic size (cM) of chromosomes (Figure 3C-F).

## Discussion

A whole genome SNP-based linkage map for the turkey is presented with 531 markers dispersed over 28 linkage groups and a total map length of 2324 cM. The total map length in the present study was slightly higher than that described by Reed et al. [3]. This difference in length is probably caused by coverage of three additional turkey chromosomes (MGA20, 24 and MGA25) along with the utilization of 69% higher number of markers in the present study, likely to be covering a larger proportion of the turkey genome. Matching the turkey linkage groups with the chicken physical map identified a map for each of the syntenic groups/chromosomes described by Griffin et al. [2]. The comparison of the syntenic chromosomes between turkey and chicken showed that the genetic lengths of turkey chromosomes were very similar to the estimated genetic length of the chicken chromosomes (Table 1). When comparing the turkey genetic linkage map and chicken physical map with respect to the order of markers across the chromosomes, some of the chromosomes (MGA14, 21, 25, 26 and MGAZ) showed complete conservation in the order of markers whereas others showed limited variation. The conservation in the order of markers for the chicken chromosomes GGA12, 19, 24 and GGAZ with the syntenic turkey linkage groups was also observed by Reed et al. [3]. This high rate of concordance in the order of markers between the genomes of these two avian species is indicative of a highly conserved nature of avian genomes.

Observed interchromosomal rearrangements (Figure 1) in the present study between turkey and chicken are in agreement with the results of Griffin et al. [2]. A number of complex intrachromosomal rearrangements (inversions) were also observed between turkey and chicken. The observed large inverted regions, of nearly a complete chromosome arm on MGA10 and MGA20 in comparison to their syntenic chicken chromosome GGA8 and GGA18 (Figure 2) were also observed in a sequence based comparative study by Dalloul et al. [29]. Cytogenetic studies using chromosome painting also reported an inversion on MGA10 in comparison to the syntenic chicken chromosome GGA8 [2,13]. Our comparative linkage map of turkey and chicken does not show pericentic inversions on MGA2 and MGA3p as were reported by Griffin et al. [2] but we have observed complex rearrangements resulting in a reversed order of markers on these chromosomes (Additional file 3). Several other chromosomes, notably MGA1, 2, 5, 8, 11, 12, 13, 15, 16, 17, 19, 22, and MGA28 as well as the chromosomes that showed interchromosomal rearrangements (MGA3 and MGA6; MGA4 and MGA9) between turkey and chicken, also showed additional complex rearrangements probably involving multiple inversions or other complex rearrangements (Figure 1). A higher number of rearrangements per Mb were observed on the microchromosomes than on the macrochromosomes. The occurrence of this high number of rearrangements at the microchromosomes could be explained by the positive association of rearrangements with recombination rate [30].

Our observed low number of interchromosomal rearrangements between the chicken and turkey genomes, confirms previous results of a high degree of interchromosomal synteny in birds as seen within a number of different comparative studies of chicken with quail, duck and zebra finch [30-32]. It has been suggested that the low number of interchromosomal rearrangements during avian genome evolution is a consequence of the small amount of interspersed repeats, segmental duplications, and pseudogenes in avian genomes, which provide little opportunity for non allelic homozygous recombination [33,34]. A relatively high number of intrachromosomal rearrangements was observed in our comparative analysis of the turkey and chicken, which agrees with the findings of the sequence based comparative studies of chicken with turkey and zebra finch [26,29,30]. The relatively high number of intrachromosomal rearrangements clearly suggests that the organization of avian genomes is more prone to intrachromosomal rearrangements than previously appreciated based on chromosome banding and chromosome painting data [2].

The comparison of male vs. female maps showed differences in genetic lengths of maps. In turkey, the total male-specific map appeared to be 195 cM longer than the female specific map. However, female-specific maps for some chromosomes (MGA10, 11, 15, 16, 23, 25, 26 and MGA28) were also found to be longer than the male maps (Table 1). The longer map length in homogametic males can be explained by the Haldane-Huxley rule [35,36], which predicts that the frequency of recombination during meiosis is lower in the heterogametic sex. The smaller map lengths in turkey for some male-specific maps were found to be an exception to the Haldane-Huxley rule. However, the longer map lengths for some chromosome maps in the heterogametic sex were also found in chicken [9].

In the present study three maps i.e. the turkey genetic linkage map, the turkey physical map and the chicken physical map were compared. The discordance of turkey physical map with the turkey genetic linkage and the chicken physical map in the allocation of marker at different chromosomes could possibly be explained by the occurrence of assembly errors in the turkey genome sequence. The turkey physical map was created completely by whole genome shotgun sequencing using Roche 454 and Illumina GA2 sequence data. Inconsistencies between the turkey linkage and chicken physical maps relative to the turkey physical map are most likely a reflection of the challenge of correctly assembling a genome based on next-gen sequencing data alone. Markers that were in agreement between turkey linkage and chicken physical maps but that could not be positioned on the turkey physical map most likely reflect an uncovered genomic regions since the turkey genome sequence is known to cover around 95% of the complete genome (Turkey genome build UMD 2.01).

In general, higher recombination rates and higher densities of GC-rich elements were found on microchromosomes compared to macrochromosomes (Figure 3A &3B). During meiosis, at least one chiasma per bivalent chromosome is required [37], but the likelihood of chiasmata forming varies along the chromosome [38]. In turkey, recombination rate and GC rich sequences (CTCF, CCTCCCT, CTCTCCC and CCCCCCC) were found to co-vary among different chromosomes. A similar trend was also seen in human, mouse and other birds like chicken and zebra finch [9,10,20,26,37,39,40].

In the present study recombination rates were found to be correlated with CpG/Mb. In general CpG/Mb tended to increase in areas of higher recombination i.e. microchromosomes (Figure 3B). This demonstrates that in the turkey microchromosomes, high recombination rate, high amount of GC-rich sequences (CTCF, CCTCCCT, CTCTCCC and CCCCCCC) and high amount of CpG contents are all correlated (Figure 3A-F). Other studies reported that GC-rich regions in a genome had higher gene densities [41,42] and that microchromosomes had higher gene densities than the macrochromosomes [38]. The nature of the microchromosomes in birds, with their high recombination rates, high amount of GC-rich sequences, GC content and gene densities appears to be an extreme instance of a general trend.

The results for MGAZ in the analysis of recombination rate and sequence motif densities across the chromosomes, were unexpected and MGAZ appeared as outlier as seen in figure 3A-F. This outlier spot could represent a true characteristic of MGAZ but more likely results from the low marker density on this particular chromosome in our analysis. (Additional Files 2 &3).

## Conclusion

Our SNP-based genetic linkage map of turkey with 531 markers reveals a genome length of 2,324 cM. This linkage map also allowed a comparison of the genome structures of turkey and chicken, demonstrating a very high degree of conservation in chromosome structure. A relatively low number of inter-, and intrachromosomal rearrangements was observed despite these two species being separated by 40 million years of evolution.

## Authors' contributions

MLA and MAMG analyzed the data. RPMAC organized the lab work and improved the paper with suggestions and comments. MLA wrote the paper and all other authors gave suggestions and comments for the improvement of paper. All authors read and approved the final manuscript.

## Supplementary Material

Additional file 1 **Detail of SBE primers along with their primer sequences and gene accession numbers**. This file contains PCR reverse and forward primer sequences along with the SNP specific SBE primer sequence. This file also contains gene name and their accession numbers. Click here for file

Additional file 2 **Linkage and physical maps (data) of turkey chromosomes along with the physical map of syntenic chicken chromosomes**. The detail of turkey linkage and physical maps along with the chicken physical map. This file also contains the flanking sequences of SNPs used in the present studied with their genotyping status. Click here for file

Additional file 3 **Linkage maps (Figures) of turkey chromosomes showing rearrangements with syntenic chicken chromosomes**. Figures showing comparative linkage maps of turkey and chicken including all the chromosomes mentioned in the present paper. Click here for file
