# Supplementary material for: A SNP based linkage map of the turkey genome reveals multiple intrachromosomal rearrangements between the Turkey and Chicken genomes
Source: BMC Genomics. 2010 Nov 20;11:647. doi: 10.1186/1471-2164-11-647 (PMC3091770; doi:10.1186/1471-2164-11-647)
Supplement: Additional file 3 — Linkage maps (Figures) of turkey chromosomes showing rearrangements with syntenic chicken chromosomes. Figures showing comparative linkage maps of turkey and chicken including all the chromosomes mentioned in the present paper. [file 1471-2164-11-647-S3.PDF]

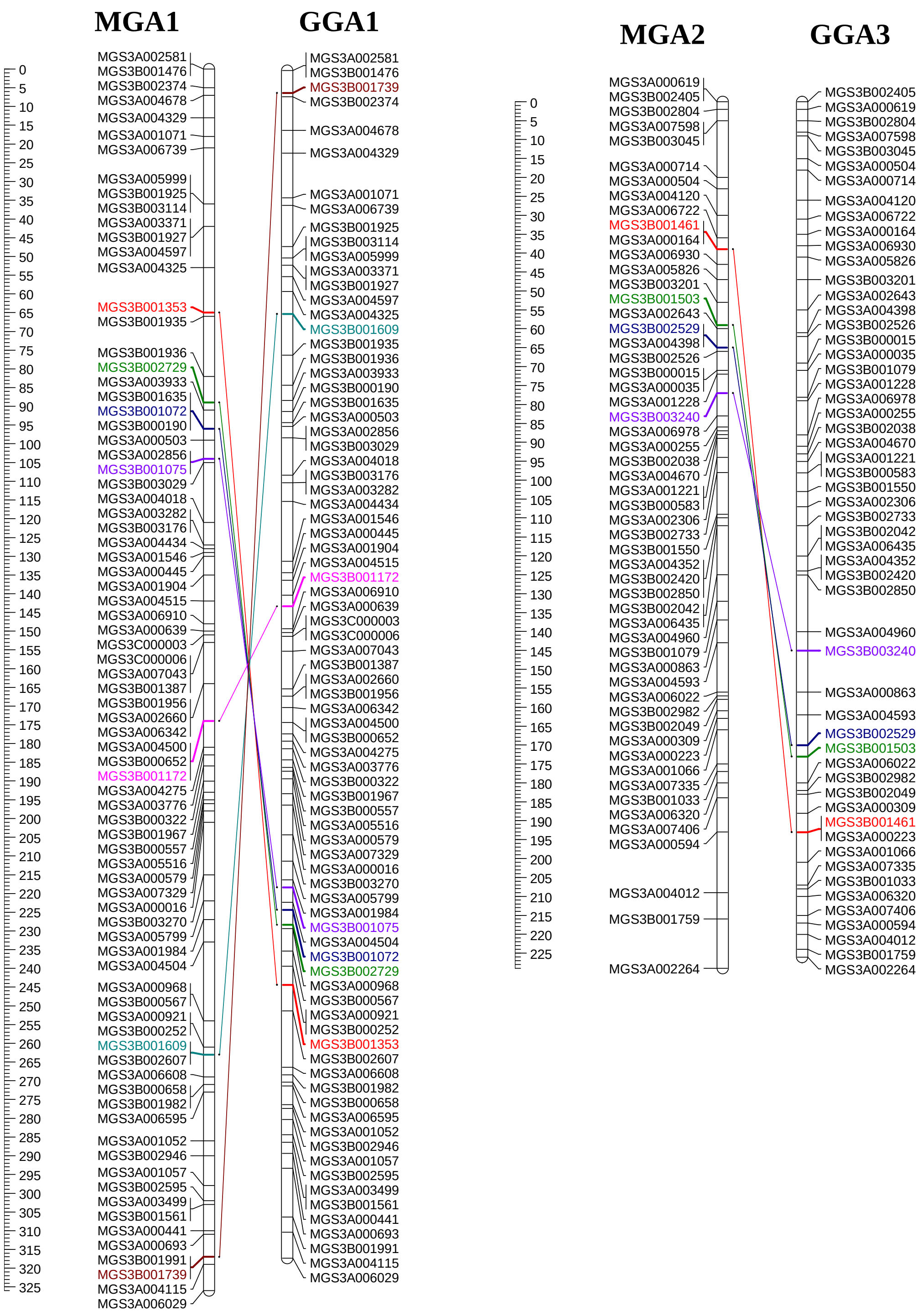
MGA1
GGA1
MGA2
GGA3
MGS3A002581
MGS3B001476
MGS3B002374
MGS3A004678
MGS3A004329
MGS3A001071
MGS3A006739
MGS3A005999
MGS3B001925
MGS3B003114
MGS3A003371
MGS3B001927
MGS3A004597
MGS3A004325
MGS3B001353
MGS3B001935
MGS3B001936
MGS3B002729
MGS3A003933
MGS3B001635
MGS3B001072
MGS3B000190
MGS3A000503
MGS3A002856
MGS3B001075
MGS3B003029
MGS3A004018
MGS3A003282
MGS3B003176
MGS3A004434
MGS3A001546
MGS3A000445
MGS3A001904
MGS3A004515
MGS3A006910
MGS3A000639
MGS3C000003
MGS3C000006
MGS3A007043
MGS3B001387
MGS3B001956
MGS3A002660
MGS3A006342
MGS3A004500
MGS3B000652
MGS3B001172
MGS3A004275
MGS3A003776
MGS3B000322
MGS3B001967
MGS3B000557
MGS3A005516
MGS3A000579
MGS3A007329
MGS3A000016
MGS3B003270
MGS3A005799
MGS3A001984
MGS3A004504
MGS3A000968
MGS3B000567
MGS3A000921
MGS3B000252
MGS3B001609
MGS3B002607
MGS3A006608
MGS3B000658
MGS3B001982
MGS3A006595
MGS3A001052
MGS3B002946
MGS3A001057
MGS3B002595
MGS3A003499
MGS3B001561
MGS3A000441
MGS3A000693
MGS3B001991
MGS3B001739
MGS3A004115
MGS3A006029
MGS3A000619
MGS3B002405
MGS3B002804
MGS3A007598
MGS3B003045
MGS3A000714
MGS3A000504
MGS3A004120
MGS3A006722
MGS3B001461
MGS3A000164
MGS3A006930
MGS3A005826
MGS3B003201
MGS3B001503
MGS3A002643
MGS3B002529
MGS3A004398
MGS3B002526
MGS3B000015
MGS3A000035
MGS3A001228
MGS3B003240
MGS3A006978
MGS3A000255
MGS3B002038
MGS3A004670
MGS3A001221
MGS3B000583
MGS3A002306
MGS3B002733
MGS3B001550
MGS3A004352
MGS3B002420
MGS3B002850
MGS3B002042
MGS3A006435
MGS3A004960
MGS3B001079
MGS3A000863
MGS3A004593
MGS3A006022
MGS3B002982
MGS3B002049
MGS3A000309
MGS3A000223
MGS3A001066
MGS3A007335
MGS3B001033
MGS3A006320
MGS3A007406
MGS3A000594
MGS3A004012
MGS3B001759
MGS3A002264

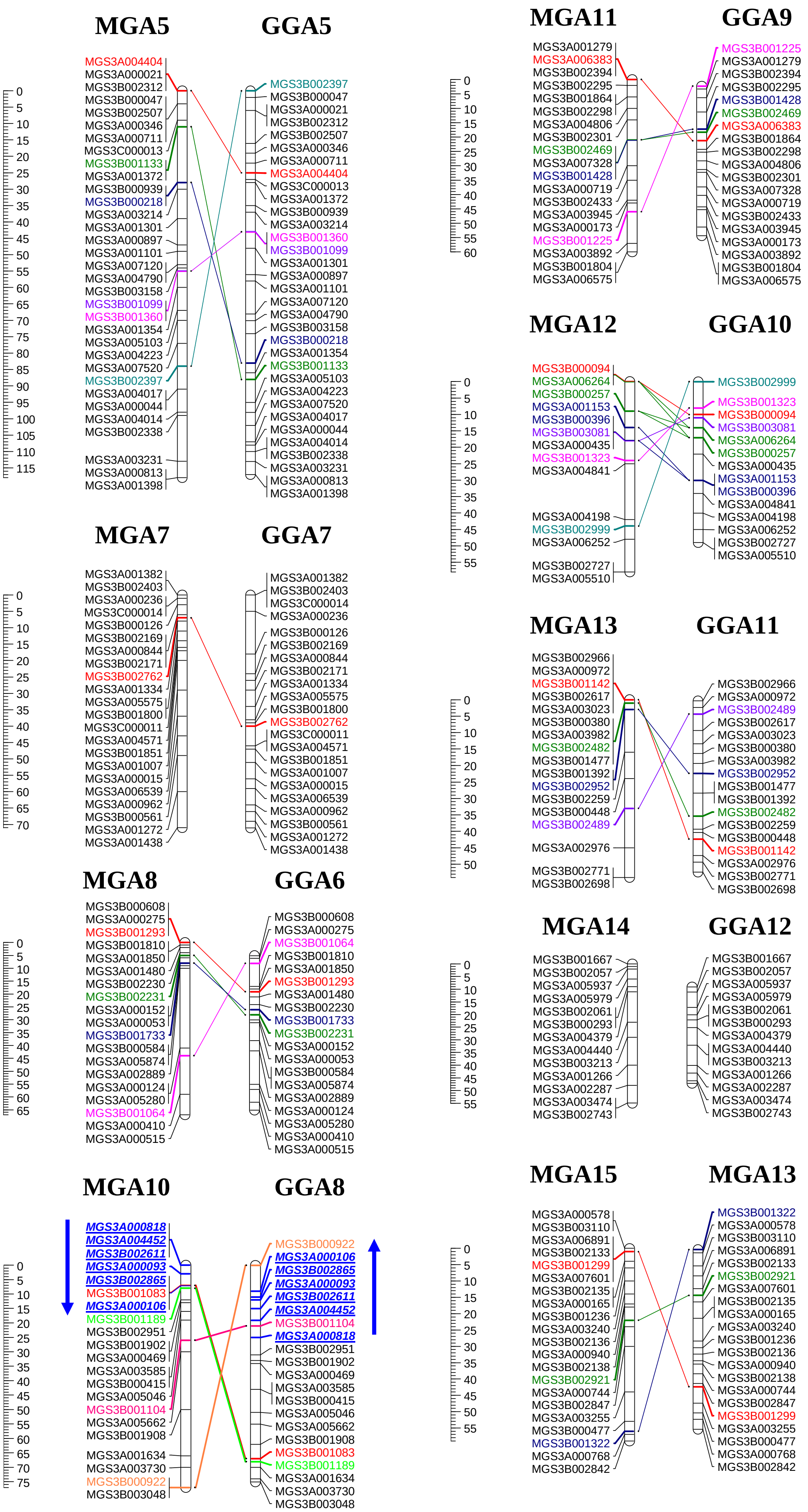

MGA5
GGA5
0 5 10 15 20 25 30 35 40 45 50 55 60 65 70 75 80 85 90 95 100 105 110 115
MGS3A004404
MGS3A000021
MGS3B002312
MGS3B000047
MGS3B002507
MGS3A000346
MGS3A000711
MGS3C000013
MGS3B001133
MGS3A001372
MGS3B000939
MGS3B000218
MGS3A003214
MGS3A001301
MGS3A000897
MGS3A001101
MGS3A007120
MGS3A004790
MGS3B003158
MGS3B001099
MGS3B001360
MGS3A001354
MGS3A005103
MGS3A004223
MGS3A007520
MGS3B002397
MGS3A004017
MGS3A000044
MGS3A004014
MGS3B002338
MGS3A003231
MGS3A000813
MGS3A001398
MGS3B002397
MGS3B000047
MGS3A000021
MGS3B002312
MGS3B002507
MGS3A000346
MGS3A000711
MGS3A004404
MGS3C000013
MGS3A001372
MGS3B000939
MGS3A003214
MGS3B001360
MGS3B001099
MGS3A001301
MGS3A000897
MGS3A001101
MGS3A007120
MGS3A004790
MGS3B003158
MGS3B000218
MGS3A001354
MGS3B001133
MGS3A005103
MGS3A004223
MGS3A007520
MGS3A004017
MGS3A000044
MGS3A004014
MGS3B002338
MGS3A003231
MGS3A000813
MGS3A001398
MGA7
GGA7
0 5 10 15 20 25 30 35 40 45 50 55 60 65 70
MGS3A001382
MGS3B002403
MGS3A000236
MGS3C000014
MGS3B000126
MGS3B002169
MGS3A000844
MGS3B002171
MGS3B002762
MGS3A001334
MGS3A005575
MGS3B001800
MGS3C000011
MGS3A004571
MGS3B001851
MGS3A001007
MGS3A000015
MGS3A006539
MGS3A000962
MGS3B000561
MGS3A001272
MGS3A001438
MGS3A001382
MGS3B002403
MGS3C000014
MGS3A000236
MGS3B000126
MGS3B002169
MGS3A000844
MGS3B002171
MGS3A001334
MGS3A005575
MGS3B001800
MGS3B002762
MGS3C000011
MGS3A004571
MGS3B001851
MGS3A001007
MGS3A000015
MGS3A006539
MGS3A000962
MGS3B000561
MGS3A001272
MGS3A001438
MGA8
GGA6
0 5 10 15 20 25 30 35 40 45 50 55 60 65
MGS3B000608
MGS3A000275
MGS3B001293
MGS3B001810
MGS3A001850
MGS3A001480
MGS3B002230
MGS3B002231
MGS3A000152
MGS3A000053
MGS3B001733
MGS3B000584
MGS3A005874
MGS3A002889
MGS3A000124
MGS3A005280
MGS3B001064
MGS3A000410
MGS3A000515
MGS3B000608
MGS3A000275
MGS3B001064
MGS3B001810
MGS3A001850
MGS3B001293
MGS3A001480
MGS3B002230
MGS3B001733
MGS3B002231
MGS3A000152
MGS3A000053
MGS3B000584
MGS3A005874
MGS3A002889
MGS3A000124
MGS3A005280
MGS3A000410
MGS3A000515
MGA10
GGA8
0 5 10 15 20 25 30 35 40 45 50 55 60 65 70 75
MGS3A000818
MGS3A004452
MGS3B002611
MGS3A000093
MGS3B002865
MGS3B001083
MGS3A000106
MGS3B001189
MGS3B002951
MGS3B001902
MGS3A000469
MGS3A003585
MGS3B000415
MGS3A005046
MGS3B001104
MGS3A005662
MGS3B001908
MGS3A001634
MGS3A003730
MGS3B000922
MGS3B003048
MGS3B000922
MGS3A000106
MGS3B002865
MGS3A000093
MGS3B002611
MGS3A004452
MGS3B001104
MGS3A000818
MGS3B002951
MGS3B001902
MGS3A000469
MGS3A003585
MGS3B000415
MGS3A005046
MGS3A005662
MGS3B001908
MGS3B001083
MGS3B001189
MGS3A001634
MGS3A003730
MGS3B003048
MGA11
GGA9
0 5 10 15 20 25 30 35 40 45 50 55 60
MGS3A001279
MGS3A006383
MGS3B002394
MGS3B002295
MGS3B001864
MGS3B002298
MGS3A004806
MGS3B002301
MGS3B002469
MGS3A007328
MGS3B001428
MGS3A000719
MGS3B002433
MGS3A003945
MGS3A000173
MGS3B001225
MGS3A003892
MGS3B001804
MGS3A006575
MGS3B001225
MGS3A001279
MGS3B002394
MGS3B002295
MGS3B001428
MGS3B002469
MGS3A006383
MGS3B001864
MGS3B002298
MGS3A004806
MGS3B002301
MGS3A007328
MGS3A000719
MGS3B002433
MGS3A003945
MGS3A000173
MGS3A003892
MGS3B001804
MGS3A006575
MGA12
GGA10
0 5 10 15 20 25 30 35 40 45 50 55
MGS3B000094
MGS3A006264
MGS3B000257
MGS3A001153
MGS3B000396
MGS3B003081
MGS3A000435
MGS3B001323
MGS3A004841
MGS3A004198
MGS3B002999
MGS3A006252
MGS3B002727
MGS3A005510
MGS3B002999
MGS3B001323
MGS3B000094
MGS3B003081
MGS3A006264
MGS3B000257
MGS3A000435
MGS3A001153
MGS3B000396
MGS3A004841
MGS3A004198
MGS3A006252
MGS3B002727
MGS3A005510
MGA13
GGA11
0 5 10 15 20 25 30 35 40 45 50
MGS3B002966
MGS3A000972
MGS3B001142
MGS3B002617
MGS3A003023
MGS3B000380
MGS3A003982
MGS3B002482
MGS3B001477
MGS3B001392
MGS3B002952
MGS3B002259
MGS3B000448
MGS3B002489
MGS3A002976
MGS3B002771
MGS3B002698
MGS3B002966
MGS3A000972
MGS3B002489
MGS3B002617
MGS3A003023
MGS3B000380
MGS3A003982
MGS3B002952
MGS3B001477
MGS3B001392
MGS3B002482
MGS3B002259
MGS3B000448
MGS3B001142
MGS3A002976
MGS3B002771
MGS3B002698
MGA14
GGA12
0 5 10 15 20 25 30 35 40 45 50 55
MGS3B001667
MGS3B002057
MGS3A005937
MGS3A005979
MGS3B002061
MGS3B000293
MGS3A004379
MGS3A004440
MGS3B003213
MGS3A001266
MGS3A002287
MGS3A003474
MGS3B002743
MGS3B001667
MGS3B002057
MGS3A005937
MGS3A005979
MGS3B002061
MGS3B000293
MGS3A004379
MGS3A004440
MGS3B003213
MGS3A001266
MGS3A002287
MGS3A003474
MGS3B002743
MGA15
MGA13
0 5 10 15 20 25 30 35 40 45 50 55
MGS3A000578
MGS3B003110
MGS3A006891
MGS3B002133
MGS3B001299
MGS3A007601
MGS3B002135
MGS3A000165
MGS3B001236
MGS3A003240
MGS3B002136
MGS3A000940
MGS3B002138
MGS3B002921
MGS3A000744
MGS3B002847
MGS3A003255
MGS3B000477
MGS3B001322
MGS3A000768
MGS3B002842
MGS3B001322
MGS3A000578
MGS3B003110
MGS3A006891
MGS3B002133
MGS3B002921
MGS3A007601
MGS3B002135
MGS3A000165
MGS3A003240
MGS3B001236
MGS3B002136
MGS3A000940
MGS3B002138
MGS3A000744
MGS3B002847
MGS3B001299
MGS3A003255
MGS3B000477
MGS3A000768
MGS3B002842

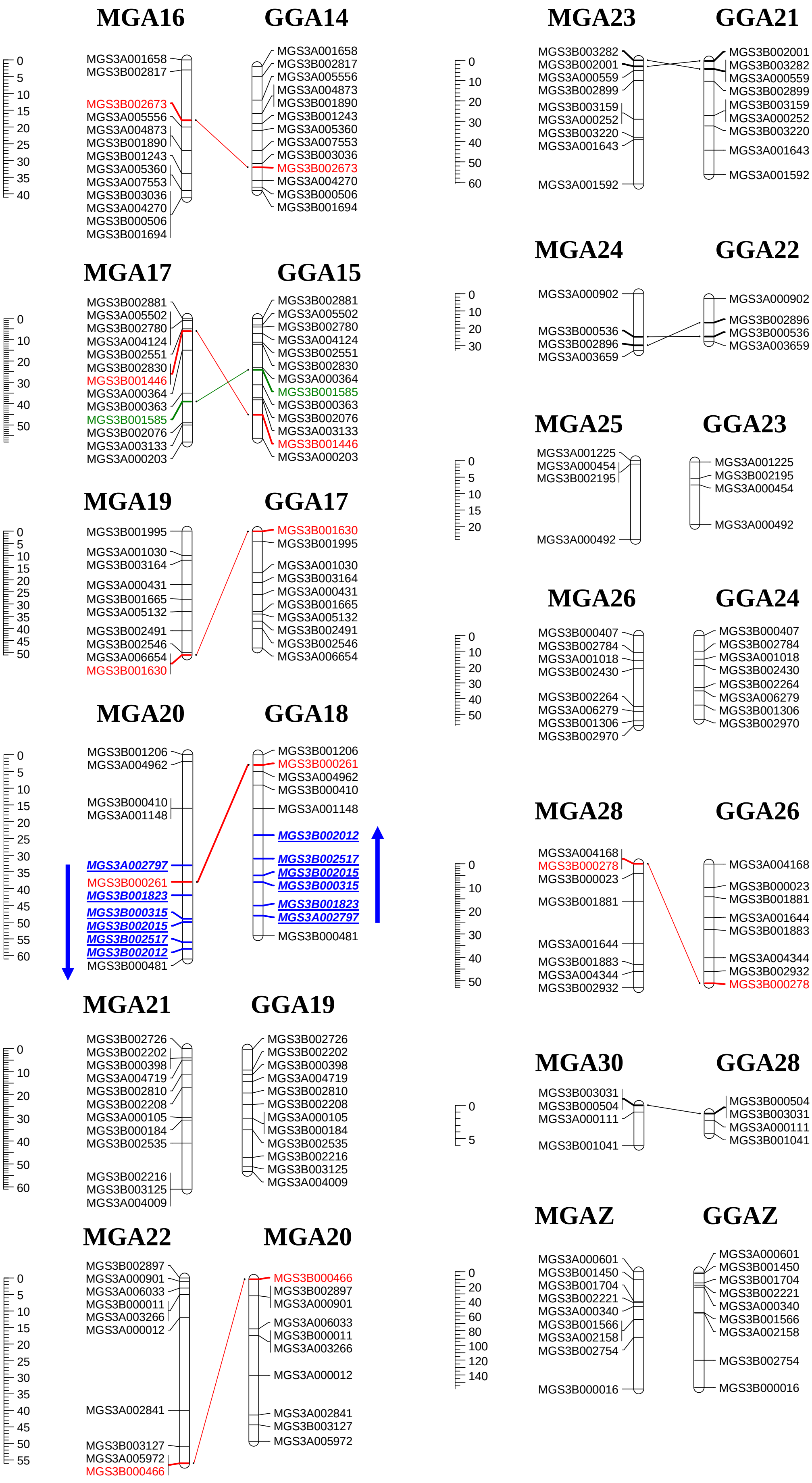

MGA16
GGA14
0 5 10 15 20 25 30 35 40
MGS3A001658
MGS3B002817
MGS3B002673
MGS3A005556
MGS3A004873
MGS3B001890
MGS3B001243
MGS3A005360
MGS3A007553
MGS3B003036
MGS3A004270
MGS3B000506
MGS3B001694
MGA17
GGA15
0 10 20 30 40 50
MGS3B002881
MGS3A005502
MGS3B002780
MGS3A004124
MGS3B002551
MGS3B002830
MGS3B001446
MGS3A000364
MGS3B000363
MGS3B001585
MGS3B002076
MGS3A003133
MGS3A000203
MGA19
GGA17
0 5 10 15 20 25 30 35 40 45 50
MGS3B001995
MGS3A001030
MGS3B003164
MGS3A000431
MGS3B001665
MGS3A005132
MGS3B002491
MGS3B002546
MGS3A006654
MGS3B001630
MGA20
GGA18
0 5 10 15 20 25 30 35 40 45 50 55 60
MGS3B001206
MGS3A004962
MGS3B000410
MGS3A001148
MGS3A002797
MGS3B000261
MGS3B001823
MGS3B000315
MGS3B002015
MGS3B002517
MGS3B002012
MGS3B000481
MGA21
GGA19
0 10 20 30 40 50 60
MGS3B002726
MGS3B002202
MGS3B000398
MGS3A004719
MGS3B002810
MGS3B002208
MGS3A000105
MGS3B000184
MGS3B002535
MGS3B002216
MGS3B003125
MGS3A004009
MGA22
MGA20
0 5 10 15 20 25 30 35 40 45 50 55
MGS3B002897
MGS3A000901
MGS3A006033
MGS3B000011
MGS3A003266
MGS3A000012
MGS3A002841
MGS3B003127
MGS3A005972
MGS3B000466
MGA23
GGA21
0 10 20 30 40 50 60
MGS3B003282
MGS3B002001
MGS3A000559
MGS3B002899
MGS3B003159
MGS3A000252
MGS3B003220
MGS3A001643
MGS3A001592
MGA24
GGA22
0 10 20 30
MGS3A000902
MGS3B000536
MGS3B002896
MGS3A003659
MGA25
GGA23
0 5 10 15 20
MGS3A001225
MGS3A000454
MGS3B002195
MGS3A000492
MGA26
GGA24
0 10 20 30 40 50
MGS3B000407
MGS3B002784
MGS3A001018
MGS3B002430
MGS3B002264
MGS3A006279
MGS3B001306
MGS3B002970
MGA28
GGA26
0 10 20 30 40 50
MGS3A004168
MGS3B000278
MGS3B000023
MGS3B001881
MGS3A001644
MGS3B001883
MGS3A004344
MGS3B002932
MGA30
GGA28
0 5
MGS3B003031
MGS3B000504
MGS3A000111
MGS3B001041
MGAZ
GGAZ
0 20 40 60 80 100 120 140
MGS3A000601
MGS3B001450
MGS3B001704
MGS3B002221
MGS3A000340
MGS3B001566
MGS3A002158
MGS3B002754
MGS3B000016

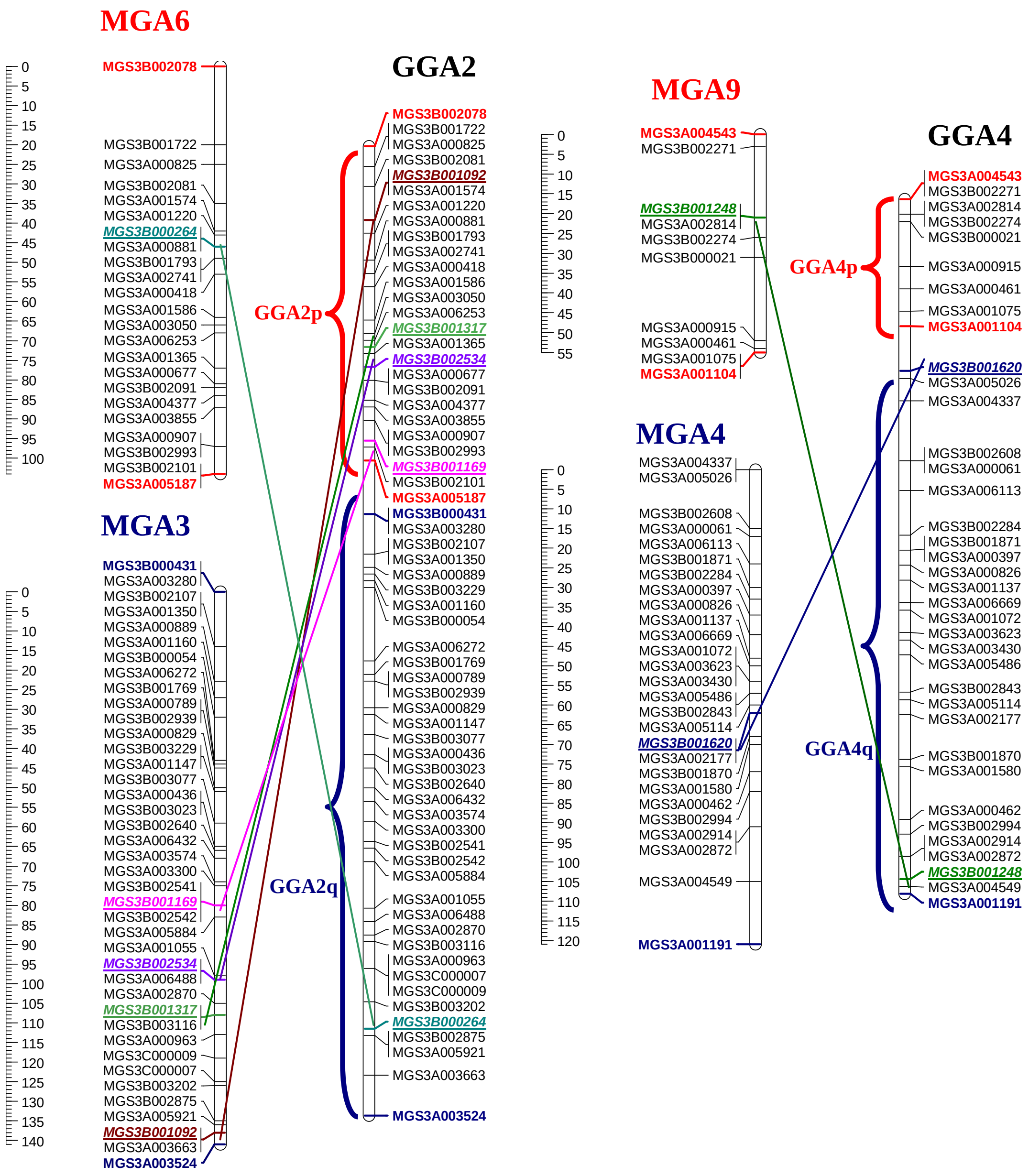
MGA6
MGS3B002078
MGS3B001722
MGS3A000825
MGS3B002081
MGS3A001574
MGS3A001220
MGS3B000264
MGS3A000881
MGS3B001793
MGS3A002741
MGS3A000418
MGS3A001586
MGS3A003050
MGS3A006253
MGS3A001365
MGS3A000677
MGS3B002091
MGS3A004377
MGS3A003855
MGS3A000907
MGS3B002993
MGS3B002101
MGS3A005187
MGA3
MGS3B000431
MGS3A003280
MGS3B002107
MGS3A001350
MGS3A000889
MGS3A001160
MGS3B000054
MGS3A006272
MGS3B001769
MGS3A000789
MGS3B002939
MGS3A000829
MGS3B003229
MGS3A001147
MGS3B003077
MGS3A000436
MGS3B003023
MGS3B002640
MGS3A006432
MGS3A003574
MGS3A003300
MGS3B002541
MGS3B001169
MGS3B002542
MGS3A005884
MGS3A001055
MGS3B002534
MGS3A006488
MGS3A002870
MGS3B001317
MGS3B003116
MGS3A000963
MGS3C000009
MGS3C000007
MGS3B003202
MGS3B002875
MGS3A005921
MGS3B001092
MGS3A003663
MGS3A003524
GGA2
GGA2p
GGA2q
MGS3B002078
MGS3B001722
MGS3A000825
MGS3B002081
MGS3B001092
MGS3A001574
MGS3A001220
MGS3A000881
MGS3B001793
MGS3A002741
MGS3A000418
MGS3A001586
MGS3A003050
MGS3A006253
MGS3B001317
MGS3A001365
MGS3B002534
MGS3A000677
MGS3B002091
MGS3A004377
MGS3A003855
MGS3A000907
MGS3B002993
MGS3B001169
MGS3B002101
MGS3A005187
MGS3B000431
MGS3A003280
MGS3B002107
MGS3A001350
MGS3A000889
MGS3B003229
MGS3A001160
MGS3B000054
MGS3A006272
MGS3B001769
MGS3A000789
MGS3B002939
MGS3A000829
MGS3A001147
MGS3B003077
MGS3A000436
MGS3B003023
MGS3B002640
MGS3A006432
MGS3A003574
MGS3A003300
MGS3B002541
MGS3B002542
MGS3A005884
MGS3A001055
MGS3A006488
MGS3A002870
MGS3B003116
MGS3A000963
MGS3C000007
MGS3C000009
MGS3B003202
MGS3B000264
MGS3B002875
MGS3A005921
MGS3A003663
MGS3A003524
MGA9
MGS3A004543
MGS3B002271
MGS3B001248
MGS3A002814
MGS3B002274
MGS3B000021
MGS3A000915
MGS3A000461
MGS3A001075
MGS3A001104
MGA4
MGS3A004337
MGS3A005026
MGS3B002608
MGS3A000061
MGS3A006113
MGS3B001871
MGS3B002284
MGS3A000397
MGS3A000826
MGS3A001137
MGS3A006669
MGS3A001072
MGS3A003623
MGS3A003430
MGS3A005486
MGS3B002843
MGS3A005114
MGS3B001620
MGS3A002177
MGS3B001870
MGS3A001580
MGS3A000462
MGS3B002994
MGS3A002914
MGS3A002872
MGS3A004549
MGS3A001191
GGA4
GGA4p
GGA4q
MGS3A004543
MGS3B002271
MGS3A002814
MGS3B002274
MGS3B000021
MGS3A000915
MGS3A000461
MGS3A001075
MGS3A001104
MGS3B001620
MGS3A005026
MGS3A004337
MGS3B002608
MGS3A000061
MGS3A006113
MGS3B002284
MGS3B001871
MGS3A000397
MGS3A000826
MGS3A001137
MGS3A006669
MGS3A001072
MGS3A003623
MGS3A003430
MGS3A005486
MGS3B002843
MGS3A005114
MGS3A002177
MGS3B001870
MGS3A001580
MGS3A000462
MGS3B002994
MGS3A002914
MGS3A002872
MGS3B001248
MGS3A004549
MGS3A001191
